# Acute liver failure following hemodialysis arteriovenous graft placement: a case report

**DOI:** 10.1186/1752-1947-4-261

**Published:** 2010-08-10

**Authors:** Zachary Z Brener, Augusto D Paiusco, Michael Bergman

**Affiliations:** 1 Department of Medicine, Beth Israel Medical Center, New York, 10003, NY, USA; 2 Department of Medicine, Hasharon-Golda, Rabin Medical Center, Petah-Tikva, 19632, Israel

## Abstract

**Introduction:**

Severe high-output cardiac failure is a serious complication of high-flow vascular access requiring immediate intervention. Ischemic hepatitis is defined as a massive increase in serum transaminase levels due to an imbalance between hepatic oxygen supply and demand in the absence of other acute causes of liver damage. It is typically preceded by hypotension, hypoxemia, or both, and occurs mostly in elderly patients with right-sided congestive heart failure.

**Case presentation:**

We report a fatal case of acute liver failure in an 84-year-old Caucasian man with high-output cardiac failure due to arteriovenous hemodialysis access. The chronological sequence of acute liver failure in the context of vascular access created two days before suggests that ischemic hepatitis was the result of high-output cardiac failure due to vascular access.

**Conclusions:**

A thorough cardiac assessment should be performed in patients with severe cardiac disease prior to placing an arteriovenous access, and arteriovenous fistula should be the preferred vascular access.

## Introduction

The chronic kidney disease (CKD) population generally has multiple risk factors and a high prevalence of cardiovascular disease. Thus, it is easy to overlook the contribution of a high-access flow to symptoms of heart failure in favor of many other possible risk factors. Severe high-output cardiac failure is a rare, but potentially fatal, complication of high-flow vascular access requiring immediate intervention [1,2]. It is much more common with prosthetic grafts than with native arteriovenous fistulas (AVF) [3].

Ischemic hepatitis is an infrequent presentation, usually associated with hypotension, especially in a presence of right-sided heart failure [4]. This case, to the best of our knowledge, is the first reported fatal case of acute liver failure in a patient with high-output cardiac failure due to arteriovenous hemodialysis access.

## Case presentation

An 84-year-old Caucasian man with a history of hypertension, cardiovascular disease and diabetic nephropathy was admitted with progressive dyspnea, abdominal distention, and pedal edema for one week. Serum K was 6.9 mmol/L, creatinine 300 μmol/L and blood urea nitrogen (BUN) 22 mmol/L. His baseline serum creatinine was 265 μmol/L. His hemoglobin was 98 g/L; international normalized ratio (INR), 1.5; partial thromboplastin time (PTT) 34.0, and liver function tests were normal. A chest radiograph showed pulmonary congestion. Our patient was treated with intravenous furosemide resulting in improvement of congestion and ascites. He remained normotensive throughout the admission and his serum creatinine decreased to 221 μmol/L. He was referred to a vascular surgeon for creation of a permanent vascular access for hemodialysis. Unfavorable vascular anatomy precluded AVF creation, and a right upper arm polytetrafluoroethylene (PTFE) graft was inserted between the brachial artery and the proximal brachial vein. After placement, no complications were noted and our patient had a palpable thrill. He was discharged home the following day. 24 hours later our patient presented with complaints of decreased urine output. Examination showed blood pressure 75/45 mmHg, clear lungs, ascites and no peripheral edema. His right arm arteriovenous graft had no palpable thrill. Serum creatinine was 326 μmol/L and BUN 27 mmol/L. Hemoglobin was 10 g/L; INR, 2.0; prothrombin time (PT), 27.5 s; PTT, 36.5 s; total bilirubin, 27.3 μmol/L; AST, 123 unit per liter (U/L); alanine aminotransferase (ALT), 88 U/L; alkaline phosphatase (ALP), 121 U/L; lactate dehydrogenase (LDH), 704 U/L. Chest radiograph was normal and electrocardiogram (EKG) showed paced rhythm without ischemic changes. Abdominal ultrasound revealed ascites, normal gallbladder with intra-hepatic and common bile ducts of normal caliber. Intravenous dopamine, fluids and antibiotics were started and our patient was admitted to the intensive care unit (ICU). On day two he remained severely oliguric and hemodialysis was started via internal jugular catheter. On day three he developed respiratory distress with fever and was intubated. Chest radiograph showed right lower lobe infiltrate. Trans-thoracic echocardiogram showed dilated left atrium, infero-septal hypokinesia, left ventricular (LV) hypertrophy, moderately reduced LV function with ejection fraction of 40% and severe tricuspid regurgitation. His blood cultures were negative. Our patient's liver function continued to decline with total bilirubin rising to 100.9 μmol/L; direct bilirubin, 92.3 μmol/L; AST, 445 U/L; ALT, 398 U/L; LDH, 1033 U/L; PT, 31.8 s; PTT, 43 s on the sixth hospital day. Our patient's condition deteriorated and he died on the seventh hospital day.

## Discussion

Ischemic hepatitis is defined as a massive increase in serum transaminase levels due to an imbalance between hepatic oxygen supply and demand in the absence of other acute causes of liver damage. It is typically preceded by hypotension, hypoxemia, or both, and occurs mostly in elderly patients with right-sided congestive heart failure [4,5]. The chronological sequence of acute liver failure in the context of vascular access created two days before suggest that ischemic hepatitis was the result of high-output cardiac failure due to vascular access. High-output cardiac failure is defined as symptoms of cardiac failure in the presence of an above-normal cardiac index (≥ 3.0 L/min/m^2^) [1]. Arteriovenous access creation results in a decreased peripheral resistance and thus increased cardiac output; the higher flow accesses have a more profound impact on the cardiac output [1,2]. This complication is more common in patients with brachiocephalic (elbow) fistulas compared with radiocephalic (forearm) fistulas, and much more common with prosthetic grafts than with native AVF [3]. Elderly patients and those with pre-existing cardiovascular disease are at high risk of developing high-output cardiac failure due to vascular access [3].

## Conclusions

A thorough cardiac assessment should be performed in patients with severe cardiac disease prior to placing an arteriovenous access, and AVF should be the preferred vascular access. We suggest that patients with intractable or worsening chronic heart failure despite medical therapy should undergo evaluation for high-output cardiac failure and may be considered for vascular access flow reduction or closure.

## Competing interests

The authors declare that they have no competing interests.

## Authors' contributions

ZZB was the principal author and was involved in the collection of data, review of literature, and preparation of the manuscript. ADP and MB were involved in the collection of literature and in editing the manuscript. All authors read and approved the manuscript.

## Consent

Written informed consent was obtained from the patient's next of kin for publication of this case report and any accompanying images. A copy of the written consent is available for review by the Editor-in-Chief of this journal.

## References

[B1] MacRae JM Pandeya S Humen DP Krivitski N Lindsay RM Arteriovenous fistula-associated high-output cardiac failure: a review of mechanisms Am J Kidney Dis 2004 43 E17 E21 10.1053/j.ajkd.2004.01.016 15112194

[B2] MacRae JM Levin A Belenkie I The cardiovascular effects of arteriovenous fistulas in chronic kidney disease: a cause for concern? Semin Dial 2006 19 349 352 10.1111/j.1525-139X.2006.00185.x 16970729

[B3] Dikow R Schwenger V Zeiger M Ritz E Do AV fistulas contribute to cardiac mortality in hemodialysis patients? Semin Dial 2002 15 14 17 10.1046/j.1525-139x.2002.00003.x 11874583

[B4] Ebert EC Hypoxic liver injury Mayo Clin Proc 2006 81 1232 1236 10.4065/81.9.1232 16970220

[B5] Seeto RK Fenn B Rockey DC Ischemic hepatitis: clinical presentation and pathogenesis Am J Med 2000 109 109 113 10.1016/S0002-9343(00)00461-7 10967151

